# Singing Mandarin? What Short-Term Memory Capacity, Basic Auditory Skills, and Musical and Singing Abilities Reveal About Learning Mandarin

**DOI:** 10.3389/fpsyg.2022.895063

**Published:** 2022-06-16

**Authors:** Markus Christiner, Julia Renner, Christine Groß, Annemarie Seither-Preisler, Jan Benner, Peter Schneider

**Affiliations:** ^1^Center for Systematic Musicology, Faculty of Arts and Humanities, University of Graz, Graz, Austria; ^2^Jazeps Vitols Latvian Academy of Music, Riga, Latvia; ^3^Department of East Asian Studies, University of Vienna, Vienna, Austria; ^4^Department of Linguistics, University of Vienna, Vienna, Austria; ^5^Department of Neuroradiology, Section Biomagnetism Heidelberg Medical School, Heidelberg, Germany; ^6^Department of Neurology, Section Biomagnetism Heidelberg Medical School, Heidelberg, Germany

**Keywords:** singing ability, fundamental and spectral listener, tone frequency, Mandarin, musical ability, short-term memory

## Abstract

Learning Mandarin has become increasingly important in the Western world but is rather difficult to be learnt by speakers of non-tone languages. Since tone language learning requires very precise tonal ability, we set out to test whether musical skills, musical status, singing ability, singing behavior during childhood, basic auditory skills, and short-term memory ability contribute to individual differences in Mandarin performance. Therefore, we developed Mandarin tone discrimination and pronunciation tasks to assess individual differences in adult participants’ (*N* = 109) tone language ability. Results revealed that short-term memory capacity, singing ability, pitch perception preferences, and tone frequency (high vs. low tones) were the most important predictors, which explained individual differences in the Mandarin performances of our participants. Therefore, it can be concluded that training of basic auditory skills, musical training including singing should be integrated in the educational setting for speakers of non-tone languages who learn tone languages such as Mandarin.

## Introduction

**Figure 1:**
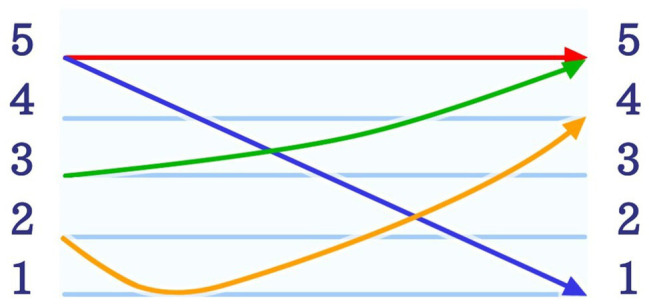
The figure illustrates the tone chart of the five Mandarin tones. According to the traditional tone chart, the red line shows the first tone which is described as a “high level tone” (5-5). Furthermore, the green line represents the second tone which is defined as the “(high) rising tone” (3-5), while the orange line shows the third tone, the “falling-rising tone” (2-1-4). Finally, the fourth tone determined as a “falling tone” (5-1) is shown by the blue line (Chao, 1965).

Mandarin is not only one of the most widely spoken languages but also by far the most widely spoken tone language in the world. Mandarin appears to be rather difficult to be learnt by speakers of non-tone languages (Christiner et al., 2018). This could be related to the fact that in tone languages, such as Mandarin, tones can denote semantic change at the lexical level. In Mandarin, every stressed/full syllable carries a syllable tone; in weak(−stressed) syllables the tone becomes neutralized (Chen, 1984). Mandarin has four different tones, which are, theoretically speaking, fixed/invariant syllable tones and one, so-called “neutral tone,” which varies according to the preceding syllable tone (Chen, 1984). Mandarin tones are often illustrated with a pitch chart (Chao, 1930), which locates the four tones within a five-level tone scale. Level 1 represents the lowest pitch and level 5 the highest as illustrated in Figure 1. According to the traditional tone chart, the first tone can be described as a “high level tone” (5-5), the second tone as a “(high) rising tone” (3-5), the third tone as a “falling-rising tone” (2-1-4), and the fourth tone as a “falling tone” (5-1; Chao, 1965). To date, this presentation of Mandarin tones is still widely accepted. The analysis of speech data, however, showed that particularly the third tone is not accurately represented within the traditional tone chart. The rising part (1-4) is normally neglected in multisyllabic expressions, leading to the reconceptualization of the third tone as a “low dipping” tone (2-1; Lin, 1985) or a low tone without the rising part. In comparison to the four full syllable tones, the neutral tone does not have a fixed tone contour. It therefore is not represented within the chart (see Figure 1). It is shorter in length and varies according to the preceding syllable. This phenomenon, also referred to as “Tone-Sandhi” (Wang, 1967; Chen, 2000) also applies to full tones, especially the third tone; however, its variation is less complex. Due to these circumstances, the neutral tone was excluded in this study. Since Mandarin requires precise ability to distinguish tones, the impact of musical ability on the acquisition of tone languages has gained increasing importance (Han et al., 2019).

## Music and Language Acquisition

Music and language share a set of comparable features and are both based on hierarchical structures (Jackendoff and Lerdahl, 2006). They consist of tonal properties and temporal features, which is a fundamental reason why overlapping features of both, language and music, have been intensively studied over the past 2 decades. For acquiring foreign languages, for taking up musical instruments, as well as for learning to sing, individuals need to be responsive to perceive and to reproduce the input they receive. Consequently, individual differences require considering perceptual and productive domains in language and music which put emphasis on similar, but also on different abilities. For instance, research on individual differences in language pronunciation skills has shown that both vocalists and instrumentalists outperformed non-musicians (Christiner and Reiterer, 2015). However, vocalists, who typically possess enhanced vocal flexibility and refined vocal motor skills, were still better in the pronunciation of unfamiliar languages than the instrumentalists (Christiner and Reiterer, 2019). On the other hand, more elaborate music and speech perceptions skills have been noted for professional instrumentalists who outperformed vocalists in a follow-up study (Christiner, 2020). Consequently, in order to give a more holistic impression of individual differences in music or language capacities, assessments should consider both dimensions production and perception. We therefore included language and music measures, which focus on perception and production tasks in order to relate these measures to Mandarin capacity.

### Neurophysiology of Music and Language

Neurophysiological research suggests that neural processing of language and music is shared to some extent, since acoustic signals of speech and music show similarities in temporal and spectral complexity (Schön et al., 2004; Ding et al., 2017). Therefore, it has been suggested that musical training reorganizes common neural circuits which not only improves musical performance but also language functions (Intartaglia et al., 2017). Positive transfer from music to language has particularly been found for aspects, which are related to phonetic ability. Musicians are generally said to be better in neural processing of non-native lexical tones (Alexander et al., 2005). Musical training has been found to enhance the neural processing of speech (Besson et al., 2011; Parbery-Clark et al., 2012; Intartaglia et al., 2017) while musical aptitude has been associated with enhanced duration speech perception (Chobert et al., 2014) and speech segmentation ability (François et al., 2013). Neurophysiological research leaves no doubt that musical capacity and musical training have positive effect on language functions.

### Cognitive-Behavioral Components of Music and Language Acquisition

Pitch is one of the most prominent features of music and language (Liu and Kager, 2017). Research has shown that musical training improves pitch discrimination ability in speech (Moreno, 2009). The ability to discriminate high vs. low tones was related to speech perception aptitude and to the number of foreign languages participants mastered (Christiner et al., 2022). Languages in which tones determine semantic information require high tonal ability. Research contrasting tone and non-tone language speakers has shown that tone language speakers possess enhanced pitch memory, show higher tonal skills and improved pitch processing ability compared to non-tone language speakers (Bidelman et al., 2013; Christiner and Reiterer, 2019). In another study, the ability to identify Mandarin tones was assessed in English speaking musicians and non-musicians. The findings revealed that musical training facilitated lexical tone identification (Lee and Hung, 2008) and the learning of Mandarin in general (Han et al., 2019). Other researchers presented similar findings and outlined that piano playing enhances processing of pitch which in turn improved word discrimination ability in Mandarin (Nan et al., 2018). Since research has shown that musical training facilitates language functions, we also wanted to address musical tonal aptitude and musical status of our participants in the research design.

While there is no doubt that musical ability improves tone language learning, individual differences in how languages are perceived may also play an important role for how well languages are performed. Recent research has shown that individuals who perceive natural languages to be more melodic than others also retrieve and pronounce these languages more accurately (Christiner et al., 2021). In addition, the researchers’ findings indicated that the high melodic language perceivers performed significantly better than the low melodic language perceivers in all typologically different languages (Christiner et al., 2021). Individual listening types can also be found in the musical domain. Most acoustic signals including the voice and the sound of music are composed of one fundamental and multiple integer harmonics (Schneider and Wengenroth, 2009). Therefore, two main dimensions have been recognized: First the fundamental pitch and second spectral pitches derived from the frequency components (Schneider et al., 2005; Seither-Preisler et al., 2007; Preisler et al., 2011). In general, there are two complementary types of listeners: “fundamental” and “spectral” listeners. What influences whether individuals are “fundamental” or “spectral” listeners is not entirely understood. Potential explanations could be genetic dispositions, but also musical practice has been shown to induce a perceptual shift from spectral toward holistic listeners (Seither-Preisler et al., 2007). Pitch perception preferences could be related to choices for musical instruments. For example, in a study about individual differences on preferences for musical instruments “[f]undamental pitch listeners played predominantly percussive or high-pitched instruments, whereas spectral pitch listeners preferred lower-pitch melodic instruments and singing” (Schneider et al., 2005, 390). As the melodic perception of unfamiliar languages had also an impact on how well languages were imitated, we also wanted to assess whether pitch perception preferences, i.e., one of the two listening types (e.g., “fundamental” or “spectral” listeners) perform better in any of our language measures. Besides pitch, rhythm is the second prominent feature which plays a crucial role in language and music. The rhythmic components of music and speech are mainly involved in how both faculties are organized. Music is characterized by a regular timed beat to which one can synchronize with periodic movements (Patel, 2007). In language, the rhythmic component facilitates that speech sounds are grouped into meaningful units. Foreign language learners often fail to understand languages as they do not master differentiating when words begin or end in a sequence of spoken language (Patel, 2007). Thus the rhythm of language does not only provide crucial information about the language phonology, but also about the syntax and semantics of phrases and sentences (Jackendoff and Lerdahl, 2006). It is therefore not surprising that musical studies have shown that rhythmic and duration abilities predict the ability to segment speech (François et al., 2013; Chobert et al., 2014). Therefore, assessing musical parameters and its relationship to language functions should also include rhythmic musical measures.

Assessing musical performance is easily achieved by using familiar song singing tasks, since they can be targeted at both musicians and non-musicians (Christiner, 2020). In particular, singing has been associated with pronunciation skills. Researchers have shown that the ability to memorize new vocabulary of adult language learners improves when words are sung (Ludke et al., 2014). The same is also true when children sing new vocabulary (Thiessen and Saffran, 2009) and a very recent study has shown that singing to infants has a positive impact on vocabulary building in later ages (Franco et al., 2021). These studies assume that the singing of new words serves as mnemonic with which new utterances are better and more easily stored in the long-term memory (Gordon et al., 2010). Beside singing as a tool which facilitates language acquisition processes, psychological and psycholinguistic research has focused on analyzing whether singing capacity facilitates language ability (Christiner, 2018; Christiner et al., 2018, 2021, 2022; Coumel et al., 2019). These studies provided evidence that the relationship between singing ability and language pronunciation has its roots in enhanced vocal-motor skills. Singers outperformed non-musicians in language pronunciation tasks (Christiner and Reiterer, 2015) as well as evidence has also been provided that the amount of singing during childhood influences both singing capacity and the ability to acquire foreign language pronunciation later in adulthood (Christiner et al., 2022). The positive relationship between singing ability and language pronunciation has been replicated for all ages. Therefore, we also wanted to assess whether singing ability as well as singing behavior during childhood also contributes to Mandarin performances.

Short-term memory (STM) capacity as measured by digit spans or non-word spans is one of the most important predictors for individual differences in the ability to acquire new languages (Dörnyei, 2005; Baddeley, 2010). Primarily, language learning processes such as speech production, reading comprehension, and vocabulary learning have been related to STM capacity (Gathercole and Baddeley, 1990, 1993; Christiner, 2018; Christiner et al., 2018). The phonological loop is most essential for language processes. It consists of two main aspects, the first being a kind of phonological store in which memory traces are held before they fade, and the second, the verbal or subvocal rehearsal mechanisms that allow decaying memory traces to be refreshed (Baddeley, 2010). Therefore, the longer the words, the more slowly they are rehearsed (Thorn and Gathercole, 2001). This increases the chance that words get lost in the phonological store (Baddeley et al., 1984). Cross-linguistic comparisons of digit span testing have shown that the shorter the names for the digits are, the higher the number of items is that can be repeated (Thorn and Gathercole, 2001). Typical tasks that measure the STM are forward span tasks where for instance numbers, dissimilar or simple words need to be recalled in a correct serial order by writing them down (Engle et al., 1999), or non-word repetition which measures the (phonological) STM (Gathercole, 2006). This may be one fundamental reason why one of our STM measures, the forward digit span, is also highly interrelated with one of the language measures as used in this study such as language pronunciation tasks of unfamiliar language stimuli. For the backward counterpart, participants are usually instructed to repeat digits or words in reversed order (Engle et al., 1999). Presumably backward spans focus more on controlled attention which makes them more of a hybrid task, but still more evidence has been provided that they may be best categorized as STM tasks. A factor analysis revealed that both forward and backward tasks are components of the same factor (Engle et al., 1999; Christiner, 2020), which could lead to the interpretation that backward spans demand a “mental transformation” as there is not a new stimulus being imposed (Conway et al., 2005). In previous research, we have noted that digit forward spans always yielded stronger relationships to language measures such as pronunciation tasks or non-word spans than backward spans (Christiner, 2020; Christiner et al., 2021). This may be the case since forward digit and forward language spans require many similar cognitive abilities which are why we also treat STM capacity as a covariate in the later parts.

Whether there is a “tonal loop” in music as an equivalent of the phonological loop for language capacity is not entirely understood. Early research speculated about the existence of a separate storage for tonal and speech material (Salamé and Baddeley, 1989), while more recently shared processing and shared neuronal networks for musical and verbal sounds have been reported (Koelsch et al., 2009; Williamson et al., 2010). This may be one reason why STM capacity is associated with enhanced language and musical capacities.

The findings of the aforementioned studies suggest that beside STM ability, musical capacities improve language functions. This reflects what current research suggests: a strong relationship between musical and language abilities. Despite the many findings which outlined relationships between musical ability and language capacity, several aspects need to be explored and addressed in more detail. Language typology is a crucial factor, and it can be suggested that tone languages have a lot in common with music which is why musical abilities seem to facilitate tone language acquisition processes. Therefore, we used music measures of previous research and developed new Mandarin language tasks in order to examine their relationship. While research has already shown a relationship between musical status and pitch perception ability, fewer research has focused on whether rhythmic ability also predict tone language capacity. In addition, there is no research available which has focused on whether one of the two complementary listener types, “fundamental” and “spectral” listeners, are benefitted in tone language acquisition. Furthermore, while singing ability and singing behavior during childhood has been related to foreign language pronunciation tasks, we also wanted to examine whether we could detect associations between one of the singing variables and the Mandarin discrimination and/or the Mandarin syllable recognition task. To address our research questions, we used the newly developed Mandarin tasks, the measures of musical aptitude, singing ability, singing behavior during childhood, basic auditory skills, and STM capacity and tested musicians, amateurs, and non-musicians.

## Materials and Methods

### Participants

In this investigation, a sample of 109 adult participants was selected who were all tested for their Mandarin performance, their musical ability (music aptitude and singing ability), their STM capacity, and basic auditory skills. The participants were all German native speakers and did not speak nor had been taught in Mandarin before they were tested. This was one of the most important parameters which should facilitate that the participants all had the same prior knowledge of Mandarin. The participants spoke foreign languages, such as English, French, Spanish, Italian, Croatian, and Dutch. The age range was *M* = 24.26 *SE* = ±1.06. In this study, 51 participants were female, whereas 58 participants were male.

### Educational Status

The participants’ educational status was specified according to the educational status which had been completed at testing time. The findings have shown that 41 participants had finished the main general secondary school, 16 the technical and vocational school, 33 secondary academic high school (general qualification for university entrance), one the post-secondary non-tertiary education, two ´bachelor studies, 15 master studies, and one had a doctoral degree.

### Musical Background

Assessing the musical background was based on our previous research (Christiner et al., 2021). Participants were instructed to label themselves to be either professional musicians, amateurs, or non-musicians. Therefore, the participants received further instructions. Being a non-musician meant that they were unable to play a musical instrument. In addition, we asked the participants whether they no longer trained or no longer played a musical instrument. The latter cases were not suitable for this research and excluded from the analyses. The participants were instructed to label themselves to be amateurs, if they could play one or more musical instruments and thus played them occasionally, but not professionally. The participants were asked to label themselves to be professional musicians if they played regularly in public as members of an orchestra at least for 2 years, or studied music for three semesters, were music teachers, or showed equivalent qualifications. According to these definitions, 34 participants were non-musicians, 30 amateurs, and 45 professional musicians.

### Measuring Mandarin Ability

Individual differences in Mandarin ability were assessed by perception and production tasks. The perception tasks consisted of two different measures: a tone discrimination and a syllable tone recognition task. The production task consisted of pronunciation tasks. The collection of our sample included all four Mandarin syllables tones [the “high level tone” (5-5), the “(high) rising tone” (3-5), the “falling-rising tone” (2-1-4), and the “falling tone” (5-1)]. The “Tone-Sandhi,” the neutral tone, was excluded from the perception tasks. We did not coin separate scores for each of the four Mandarin syllable tones but used only composite scores of the three Mandarin measures which we developed.

#### Tone Discrimination Task (Mandarin D)

The tone discrimination task consisted of 18 paired samples, which were either identical or contained a change of a particular sound in the second statement (e.g., bùzhì vs. bùzhī). The length of the words and phrases of the 18 examples varied between 2 and 11 syllables. The first statement of the paired samples was separated by a pause of 1 s from the second statement played. All paired samples are introduced by a different speaker who indicates the paired sample by a number. Before the participants run the test, they receive four practicing items, where they were introduced to the task. They were instructed by the experimenter and allowed to practice as long as they understood the tasks correctly. After familiarization, they run the entire samples in a sequence.

#### Syllable Tone Recognition Task (Mandarin S)

In this condition, the participants were again listening to paired samples but had to decide in which syllable of the second statement a tonal change occurred. The syllable tone recognition task consists of 16 paired samples where a particular syllable in the second statement contains a tonal change which has to be indicated. All syllables were separated from each other and visually presented to the participants. As the two statements differed by only one tonal change, the wording and syllable structure of both statements in all conditions were the same, and just the diacritics were removed. This aimed at avoiding tonal changes that were recognized based on the visual tone representation. The length of the words and phrases of the samples vary between two and seven syllables. Like for the tone discrimination task, the participants received practicing items before they ran the entire samples in a row.

#### Mandarin Pronunciation Task (Mandarin P)

The speech production task consisted of three Mandarin phrases (spoken by native speakers) of 7, 9, and 11 syllables, which were repeated by the participants after they had listened to them for the third time. Assessing individual differences in language pronunciation has already been carried out in previous investigations and has shown high ecological validity, since it resembles a foreign language learning condition (Christiner, 2020; Christiner et al., 2021). Like in former investigations, the recordings of the participants were normalized for their loudness and rated by five Mandarin native speakers. They were introduced to evaluate how well the participants preserved the rhythmical structure, the melodic aspects of the original phrase, completeness of the sentence material, and the overall performance. Therefore, they had to give a score which ranged between 0 and 10. The four criteria were then collapsed into a single score. The interrater reliability was assessed by intra-class coefficient analysis, as provided in the supplement (see Supplementary Table S1).

### Gordon’s Musical Aptitude Test

The Advanced Measures of Musical Audiation (AMMA test; Gordon, 1989) consists of rhythm and tonal discrimination tasks and measures the ability to internalize musical structures. The paired musical statements are embedded in one single test design where either rhythmic, tonal, or no changes may occur. For the tonal discrimination tasks (AMMA T), notes are modified in pitch in the condition when the melody is played for the second time. In the rhythm subtest (AMMA R), tempo, meter, or duration may be altered in the comparison condition. This aptitude test is usually targeted at university music and non-music majors and high school students. The test consists of 33 items, whereby the first 3 are familiarization tasks that were excluded from the final analysis.

### Tone Frequency and Duration

To test basic sound discrimination abilities, two subtests (tone frequency and duration) of the primary auditory threshold measure KLAWA (*Klangwahrnehmug*) were used. KLAWA is an inhouse computer-based threshold measurement. Difference limes are measured for tone frequency (“low vs. high”) and duration (“short vs. long”). Based on an “alternative-forced choice” (Jepsen et al., 2008), this method to measure individual perceptual thresholds can be used for scientific investigations to study subjective auditory processing and language development. In this computer-aided test procedure exact scientifically measured quantities [cent = 1/100 semitone for recording the pitch, and milliseconds (ms) for time measurements], the above-mentioned hearing performance was determined, which can largely vary from subject to subject (>factor 100).

In an alternative forced-choice paradigm, reference, and test tones (sinusoids) separated by an interstimulus interval of 500 ms are presented. Participants are asked to decide per mouse-click, which of the presented tones sounds higher or longer in the tone frequency subtests and which of the presented sounds are shorter or longer for the duration subtest. If the answers are correct, the differences become smaller in small steps; if the answers are incorrect, they become larger again. In this procedure, which automatically adapts to the performance of the tested subjects with increasing difficulty, the individual threshold values are finally calculated based on the convergence behavior.

### Pitch Perception Preference Test

The pitch perception preference test (Pitch PP) includes 144 different pairs of harmonic complex tones. Each tone pair consisted of two consecutive harmonic complex tones (duration 500 ms, 10-ms rise-fall time, and interstimulus interval 250 ms). Each test tone comprised two, three, or four adjacent harmonics, leaving out the fundamental frequency. Overall, the tone pairs were designed with six different upper component frequencies (293, 523, 932, 1,661, 2,960, and 5,274 Hz) chosen to be equidistant on a logarithmical frequency scale corresponding to the musical interval of a major ninth, beginning with D3 (293 Hz) up to C8 (5,274 Hz). The upper component frequency of both tones in each tone pair was identical to minimize the perception of edge pitch. All stimuli were presented binaurally in pseudorandomized order using Hammerfall DSP Multiface System with a stimulus level of 50 dB nSL to avoid the interfering superposition of combination tones. Each tone pair was repeated once and the next tone pair presented after a pause of 2 s. Subjects were instructed to select the predominantly perceived pitch direction or to answer according to the first, spontaneous impression. They could also indicate, if either both directions were perceived at the same time or if tones lacked a clear pitch. Test duration was 22 min. The experimental design has been described in detail in a previous study (Schneider et al., 2005).

### Singing Ability and Singing Behavior During Childhood

Singing ability was tested by a familiar song singing task “Happy Birthday,” which is usually targeted at non-professionals (Dalla Bella et al., 2007; Dalla Bella and Berkowska, 2009). This approach has been used in several previous investigations (Christiner and Reiterer, 2013; Christiner et al., 2018, 2021; Christiner, 2020). The participants were introduced to sing “Happy Birthday” as best as they could and to use a key which they found pleasurably for their own singing voice. The singing performances of the participants were rated and evaluated by singing experts (two male and two female raters), a test design which has successfully been used and tested in previous studies (Christiner, 2013; Christiner and Reiterer, 2013). The raters were introduced to their tasks and the rating criteria: melodic and rhythmic ability. The two criteria were collapsed into a single score (S total). For the interrater reliability, intraclass correlation coefficients were calculated and it was found that the ratings were reliable (see Supplementary Table S2).

We also used a multi-item scale concept, which asked for the participants’ singing behavior during childhood (S childhood) and reported how many hours the participants sang per week on average (S hours). To make sure that the participants referred to the same time period, they received further instructions. It was explained that their childhood meant before the age of 11 years since it has been suggested that the singing voice reaches around two octaves at the age of 10 (Welch et al., 2011), which is similar for adults without vocal training (Christiner, 2020). The concept consists of eight questions and has already been used previously (Christiner, 2020). The internal consistency of the concept was found to be reliable. Cronbach’s *α* = 0.79 for the eight questions. The single questions and the reliability analysis are contained in the supplement (see Supplementary Table S3).

### Short-Term Memory

In order to test the STM capacity of the participants, the Wechsler Digit Span (Wechsler, 1939) was used. This measurement consists of a backward digit span (STMB) and a forward digit span (STMF) subtest. The test was programmed online, and the stimulus was presented acoustically. The participants had to repeat a steadily increasing sequence of digits in either a forward or a backward order. The sequences of digits varied between 3 and 9 digits for the forward span and between 2 and 8 digits for the backward span subtests. Participants received two scores, one for the forward task (STMF) and one for the backward task (STMB). The score corresponded to the number of items they were able to correctly repeat, the maximum being 14. Based on previous research, mean values for the forward span usually range between values of 7 and 8 for the forward span for adult participants, while the mean values for the backward span are usually around one point lower than that of the forward span (Christiner, 2020; Christiner et al., 2021).

## Results

### Statistical Analysis

We ran correlational and regression analyses for the musical variables and the specific Mandarin measurements. In addition, we performed a MANOVA with musical status (non-musicians, amateurs, and professional musicians) as a fixed factor and the three measures of Mandarin as dependent variables. As a follow-up, we performed discriminant analyses to uncover whether the Mandarin measures differentiated between our groups. Since we were also looking at whether STM influence the relationship between Mandarin and our musical as well as basic auditory skills measures, we performed partial correlations for the variables which were correlated with Mandarin performance and STM. These are singing ability and Frequency (for examination, please consult the correlation’s Table 1). In addition, we performed an ANCOVA in which Mandarin total was the dependent variable, musical status the fixed factor, and STM (the forward span) the covariate. First, Table 2 below illustrates the descriptive statistics.

**Table 1:** Correlational analysis outlines the correlations between the variables under consideration.

	Mandarin S	Mandarin D	Mandarin P	S total	S hours	S childhood	Duration	Frequency	Pitch PP	STMF	STMB	AMMA T	AMMA R	Musical status
Mandarin total	0.688^**^	0.708^**^	0.714^**^	0.387^**^	0.036	0.044	−0.147	−0.320^**^	−0.352^**^	−0.398^**^	0.167	0.178	0.053	0.363^**^
Mandarin S		0.250^*^	0.412^**^	0.376^**^	0.060	0.061	0.000	−0.277^**^	−0.197^*^	0.209^*^	0.066	0.136	0.103	0.372^**^
Mandarin D			0.344^**^	0.234^*^	0.001	−0.034	−0.100	−0.227^*^	−0.283^**^	−0.388^**^	0.273^**^	0.215^*^	0.090	0.313^**^
Mandarin P				0.239^*^	0.001	0.269^**^	−0.159	−0.238^*^	−0.378^**^	0.301^**^	0.028	0.051	0.003	0.307^**^
S total					0.277^**^	0.302^**^	−0.074	−0.320^**^	−0.206^*^	0.310^**^	0.260^**^	0.250^**^	0.225^*^	0.429^**^
S hours						0.384^**^	0.013	0.150	−0.013	0.055	−0.054	−0.031	−0.004	−0.017
S childhood							0.057	0.091	−0.044	−0.126	0.259^**^	−0.060	−0.100	0.141
Duration								0.148	0.138	−0.120	−0.086	−0.113	−0.090	−0.133
Frequency									0.090	−0.198^*^	−0.072	−0.185	−0.180	0.311^**^
Pitch PP										−0.187	−0.106	−0.028	−0.014	−0.207^*^
STMF											0.575^**^	0.268^**^	0.241^*^	0.223^*^
STMB												0.328^**^	0.348^**^	0.106
AMMA T													0.761^**^	0.291^**^
AMMA R														0.266^**^

The following description explains the acronym definitions of the variables. Mandarin S, syllable tone discrimination task; Mandarin D, tone discrimination task; Mandarin P, pronunciation task; S total, melodic and rhythmic singing ability total; S hours, singing hours per week; S childhood, singing behavior during childhood; Duration, primary auditory threshold test—Subtest Duration; Frequency, primary auditory threshold test—Subtest Frequency; Pitch PP, pitch perception preference test; STMF, short-term memory forwards; STMB, short-term memory backwards; AMMA R, Advanced Measures of Music Audiation—rhythmic score; and AMMA T, Advanced Measures of Music Audiation—tonal score.

* Means that *p* < 0.05 (uncorrected, two-tailed).

** Indicates that *p* < 0.001 (uncorrected, two-tailed).

**Table 2:** Descriptive statistics provide the descriptives of the variables under consideration.

Variables	Mean (*M*)	Standard Error (*SE*)
Mandarin total^*^	0.03	0.07
Mandarin S	4.65	0.19
Mandarin D	13.20	0.21
Mandarin P	3.23	0.13
S total	5.99	0.12
S hours	1.89	0.25
S childhood	37.71	1.51
Duration	44.03	2.09
Frequency	26.40	1.93
Pitch PP	35.24	2.62
STMF	7.19	0.19
STMB	6.58	0.21
AMMA T	24.25	0.39
AMMA R	26.57	0.41

The following description explains the acronym definitions of the variables. Mandarin S, syllable tone discrimination task; Mandarin D, tone discrimination task; Mandarin P, pronunciation task; S total, melodic and rhythmic singing ability total; S hours, singing hours per week; S childhood, singing behavior during childhood; Duration, primary auditory threshold test—Subtest Duration; Frequency, primary auditory threshold test–Subtest Frequency; Pitch PP, pitch perception preference test; STMF, short-term memory forwards; STMB, short-term memory backwards; AMMA R, Advanced Measures of Music Audiation—rhythmic score; and AMMA T, Advanced Measures of Music Audiation—tonal score.

* Note that the Mandarin total is comprised of all three Mandarin tasks. Since the Mandarin subtests are based on different scorings, they were *z*-transformed before they were collapsed into a single score.

### Correlational Analysis

A Pearson correlational analysis was applied for the individual variables to illustrate the relationships between the Mandarin variables, STM, and the musicality measures. Table 1 illustrates the relationships between the variables.

### Regression Model for the Criterion Variable the “Mandarin Total”

Based on the findings of the correlations, we performed regression models. Before, we were assessing whether the dependent variable was normally distributed, and we therefore applied a Shapiro–Wilk test. Results have shown the Mandarin total score was normally distributed *p* = 0.86. The independent predictor variables were entered in the multiple linear regression models only if a probability of *F*-change < 0.05 was given. We used a stepwise method where the ordering of the variables is based on purely mathematical decisions. The findings revealed that four predictors, STMF, singing ability, pitch perception preference, and tone frequency could explain 33 % of the variances in the Mandarin total performance, which consists of all three tasks. Table 3 shows the results of the multiple regression. Note that we also performed multiple regressions for the three individual Mandarin tasks. The findings are provided in the Supplementary Table S4.

**Table 3:** Multiple regression models explaining the variance in Mandarin total.

Predictor	Partial correlation (pr)	*p*-value
Step 1: *R* = 0.41, *F*(1, 105) = 21.48, *p* < 0.001		
STMF	0.41	< 0.001
Step 2: *R* = 0.49, *F*(1, 104) = 10.66, *p* < 0.001		
STMF	0.33	< 0.001
S total	0.31	< 0.001
Step 3: *R* = 0.55, *F*(1, 103) = 8.19, *p* = 0.005		
STMF	0.31	< 0.001
S total	0.27	0.005
Pitch PP	−0.27	0.005
Step 4: *R* = 0.58, *F*(1, 102) = 4.43, *p* = 0.038		
STMF	0.29	< 0.002
S total	0.22	0.028
Pitch PP	−0.28	0.005
Frequency	−0.20	0.038
*Dependent variable: Mandarin total*		

This table illustrates the stepwise multiple regression models. Mandarin total is the dependent variable. The ordering of the variables is based on mathematical decisions. The following description explains the acronym definitions of the variables. STMF, short-term memory forwards; S total, melodic and rhythmic singing ability total; Pitch PP, pitch perception preference test; and Frequency, primary auditory threshold test—Subtest Frequency.

### MANOVA for Variables “Musical Status” and “Mandarin Performance”

In order to find out, whether the Mandarin performances of our participants differed in their mean values according to their musical status, we performed a MANOVA. Using Pillai’s trace, there was a significant effect of musical status and Mandarin performance *V* = 0.257, *F*(6,192) = 4.72, *p* < 0.001.

### Discriminant Analysis

**Figure 2:**
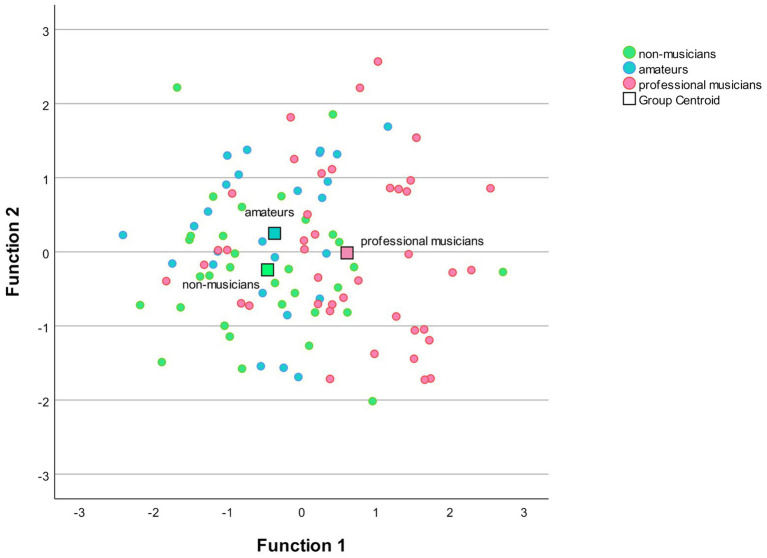
The figure illustrates the discriminant function of the three Mandarin measures. The horizontal axis represents the significant discriminant function, function 1. It discriminates the professional musicians from the amateurs and the non-musicians. The correlations between the outcome variables and the discriminant functions revealed that all three Mandarin variables load onto the first function illustrating that the professional musicians performed significantly better in Mandarin than both other groups. The vertical axis represents the second-discriminant function, function 2. Since this function was non-significant it will not be further discussed.

The MANOVA was followed by a discriminant analysis, which revealed two discriminant functions. The first explained 85.4% of the variance, canonical *R*^2^ = 0.31, whereas the second explained only 14.6%, canonical *R*^2^ = 0.04. In combination, these discriminant functions significantly discriminated the groups, Λ = 0.75, *χ*^2^(6) = 27.32, *p* < 0.001, but removing the first function indicated that the second function did not significantly differentiate the three groups Λ = 0.96, *χ*^2^(2) = 2.31, *p* = 0.51. The correlations between the considered variables and the discriminant functions revealed that the loads onto the first function were rather high for all three Mandarin variables, Mandarin S (*r* = 0.86), Mandarin P (*r* = 0.64), and Mandarin D (*r* = 0.59). When a cutoff of 0.40 was used to decide which of the standardized discriminant coefficients were large, all three Mandarin variables separated the musicians from the amateurs and the non-musicians well. The musicians had higher scores in Mandarin S (*M* = 5.69), Mandarin P (*M* = 3.70), and Mandarin D (*M* = 13.93), than the amateurs Mandarin S (*M* = 3.83), Mandarin P (*M* = 3.19), and Mandarin D (*M* = 13.07), and non-musicians Mandarin S (*M* = 4.00), Mandarin P (*M* = 2.69), and Mandarin D (*M* = 12.38). Figure 2 below illustrates the discriminant plot.

### Partial Correlations

Short-term memory, in particular the forward digit span, turned out to be an important predictor for Mandarin performance in the regression model. As introduced in section 1.3.4, forward digit spans and language measures require similar cognitive ability which is why it could be assumed that language measures and digit spans measure very similar concepts. Therefore, we ran also partial correlations for the variables which correlated with Mandarin and one of our STM ability measures. These variables were singing ability which correlated with Mandarin total and both the STMF and STMB measures as well as Frequency which correlated with Mandarin and STMF capacity.

The partial correlations reveal that when STM forward on the relationship between the singing ability and Mandarin total is controlled *r* = 0.30, *p* (two-tailed) < 0.001, their relationship diminishes but remains significant. The partial correlations reveal that when STM backward on the relationship between the singing ability and Mandarin total is controlled *r* = 0.36, *p* (two-tailed) < 0.001, their relationship diminishes but remains significant. The partial correlations reveal that when STM forward on the relationship between the Frequency and Mandarin total is controlled *r* = −0.27, *p* (two-tailed) < 0.006, their relationship diminishes but is remains significant.

### ANCOVA

We also performed an ANCOVA in which Mandarin total was the dependent variable, musical status, the fixed factor, and STM (the forward span) as the covariate. The ANCOVA revealed that the covariate, STM, was significantly related to the Mandarin total performance *F*(1,105) = 15.98, *p* < 0.001, *r* = 0.36. There was also a significant effect which could be detected for the musical status and the Mandarin performances after controlling for STM capacity *F*(2,105) = 3.72, *p* < 0.028, partial *η^2^* = 0.07.

## Discussion

We performed several different statistical analyses in order to outline the relationships between Mandarin ability and musical ability. Correlational analysis has shown that all three Mandarin subtests were related to STM ability, tone frequency, pitch perception preference, singing ability, and musical status. A regression analysis revealed that the variance of the Mandarin total performance could be explained by STM capacity, singing ability, pitch perception preference, and tone frequency. In addition, we performed a MANOVA followed by a discriminant analysis, which revealed that professional musicians were better than amateurs and non-musicians at all Mandarin conditions. Finally, we also assessed whether STM ability influences the relationship between the musical variables and the Mandarin performances and the basic auditory skills and STM capacity. Results revealed that both STM capacity and musical skills/status contribute to explaining individual differences in Mandarin performances.

### Short-Term Memory

The STM capacity as measured *via* the forward digit span was correlated to all Mandarin measures and turned out to predict the Mandarin total performance as shown in the regression model. The backward digit span was only correlated with performance in the Mandarin tone discrimination task. The finding that the forward span was a better predictor for the language tasks than the backward span has also been found in previous research (Christiner, 2020; Christiner et al., 2021). In general, we expected STM capacity to be one of the most important predictors for explaining individual differences in Mandarin performance. STM capacity is one of the most important predictors which explains individual differences in speech production, reading ability, foreign language comprehension, or vocabulary learning (Gathercole and Baddeley, 1990, 1993) and therefore has been associated with foreign language success (Dörnyei, 2005; Wen and Skehan, 2011; Wen et al., 2017). In previous research, we used mainly pronunciation tasks which are rather similar to non-word spans (Christiner, 2020; Christiner et al., 2021, 2022) which are why we expected that a positive relationship between STM capacity and Mandarin P will be found. Mandarin S and Mandarin D are measures, which were newly developed and used the first time in our studies. Mandarin D is a discrimination task in which tonal changes of an equally worded second statement need to be detected—a capacity which also requires STM capacity and is also very similar to measures of basic auditory skills (e.g., tone frequency) and musical aptitude (e.g., AMMA T). The latter both also correlated to the STM measures in this study. Mandarin S can also be seen as a task which focuses on phonetic coding ability. This has been associated with the ability to identify distinct sounds and to form associations between these sounds (Carroll, 1981). The main difference of our Mandarin S measure to already established and approved measures of phonetic coding ability such as the, the *Phonetic Script* and the *Spelling Cues* of the *Modern Language Aptitude Test* by Carroll and Sapon (1959) is that our participants had additionally to indicate a tonal change. Relationships between STM ability and coding ability have also been reported in multiple studies (e.g., Brady, 1986; Holtz, 1993).

However, STM capacity was also correlated with the musical aptitude measures and singing ability which suggests that STM capacity shows overlaps between tonal and verbal material. In early research, this notion was questioned, and it was speculated that there may be different storage components for tonal and speech material (Salamé and Baddeley, 1989). In contrast, more recently findings from brain research suggest that verbal and tonal storage and rehearsal abilities largely rely on overlapping neuronal networks (Koelsch et al., 2009). The findings of Koelsch et al. (2009) suggest overlapping or shared storage components for verbal and tonal material, which suggests that STM capacity is crucial for explaining individual differences in the performance of both music and language tasks. Since some of music variables were also related to STM capacity, we assessed whether the relationship between musical and language variables was influenced by STM capacity. Therefore, we performed partial correlations and an ANCOVA where we treated the forward STM capacity as a covariate. Results have shown that the relationship between singing ability and Mandarin performance as well as Frequency and Mandarin total performance slightly diminish when controlled for STM capacity but still remain significant. Similar results provided the ANCOVA which has shown that a significant effect could be detected for the musical status and the Mandarin performances after controlling for STM capacity. This suggests that both STM capacity and musical ability contribute to explaining individual differences in Mandarin performances.

### Production: Singing Ability and Behavior During Childhood

Singing ability is also one of the most important predictors to explain the variability in the Mandarin performance in this investigation. Singing has been found to improve the ability to memorize new vocabulary (Ludke et al., 2014; Franco et al., 2021) and the ability to imitate unfamiliar languages (Christiner and Reiterer, 2018; Christiner, 2020; Christiner et al., 2021) in children, adolescents, and adults. In this respect, two crucial elements should be discussed: melody as mnemonic and vocal-motor ability. Enhanced vocal-motor ability is required for an elaborate singing capacity, which has been identified as a predictor of individual differences in foreign language pronunciation for several times (Christiner and Reiterer, 2013). Singing behavior during childhood was also correlated with Mandarin pronunciation—a finding which has already been observed in our previous research (Christiner, 2020; Christiner et al., 2022). Singing ability as well as the amount of singing during childhood seems to enhance vocal-motor skills, sensorimotor ability, and vocal flexibility, which may be the link between singing ability and language pronunciation. Research has also shown that singing during childhood influences the ability to acquire foreign language pronunciation later in adulthood, while the same has not been found for language perception (Christiner et al., 2022).

While it has been expected that Mandarin pronunciation will be related to singing ability, the finding that the two other Mandarin tasks also correlated with singing was more surprising. In this respect, the second parameter “melody as mnemonic” should be discussed. Melody has also been ascribed to play a key role in language acquisition processes. Infants and adults do acquire new utterances much faster when they are sung (Thiessen and Saffran, 2009; Ludke et al., 2014). This may be the case since melody seems to serve as mnemonic with which new utterances are probably stored in the long-term memory (Gordon et al., 2010). Research has also noted that languages which appear to be more song-like or melodic are also better retrieved (Margulis et al., 2015; Christiner et al., 2021). Therefore, it may be assumed that individuals who perceive Mandarin to be more melodic or song-like may also perform better at all Mandarin tasks. We suggest that the singing benefit is based on enhanced sensorimotor ability and melody as mnemonic (Christiner et al., 2022).

### Perception

To recap, as opposed to non-tone language speakers, tone language speakers possess enhanced pitch memory, show higher tonal ability, and possess enhanced pitch processing ability (Bidelman et al., 2013; Nan et al., 2018; Gottfried, 2019). Tone frequency is one of the most important predictors to explain individual differences in the Mandarin ability of our tasks. Being able to differentiate the four Mandarin tones, the “high level tone,” the “(high) rising tone,” the “falling-rising tone,” and the “falling tone” requires the precise ability to discriminate low from high tones. Since our tasks consist of equally worded items, which in the different condition contained a single tonal change, we expected that tone frequency will turn out to be one of the most important predictors, while short vs. long tones (duration) played a minor role. Previous research has also outlined that individuals with better tonal ability performed better in Mandarin discrimination tasks (Gottfried, 2019). Listeners were exposed to Mandarin words that had either different or the same tones—tasks which were very similar to our Mandarin D measure. Results showed that individuals with higher musical ability and participants with professional musical background performed more accurately than non-musicians (Gottfried, 2019). Other researchers presented similar findings and outlined that piano playing enhances neural processing of pitch which in turn improved Mandarin word discrimination ability (Nan et al., 2018).

Studies have shown that musical ability was correlated with accuracy in the performance of tone-word perception and production ability (Li and DeKeyser, 2017). Interestingly, in our study, musical aptitude was less related to the Mandarin performances as only the tonal parameter correlated with the Mandarin discrimination, while the rhythmic aptitude measure was not correlated with any of the Mandarin tasks. Even though musical aptitude is generally associated with language ability, similar findings have already been observed in our previous research where eight languages were investigated. Findings revealed that only the tonal aptitude factor contributed to individual differences in foreign language capacity while the rhythmic component always failed in regression models (Christiner, 2020). On the other hand, the nature of the Mandarin tasks was designed to detect single tonal changes in sequences of language material which may be one reason why tone discrimination ability, the measurement frequency (high vs. low tones), was a better predictor than musical aptitude measures.

Interestingly, our findings have shown that Mandarin ability was enhanced in individuals who were fundamental listeners. There are two reasonable explanations. One is that musical practice may induce a shift from spectral toward fundamental pitch perception (Seither-Preisler et al., 2007). This would also be supported in the present investigation since there was also a relationship between the tendency to classify complex tones according to their fundamental pitches and musical status (see the negative correlations). Another explanation could be found in differences between fundamental pitch and spectral listeners. Whereas the former perceive the sound predominantly according to its fundamental pitch, which is a holistic feature, spectral listeners may either decompose the sound into single harmonic constituents (Schneider and Wengenroth, 2009) or perceive them in terms of global timbre (Seither-Preisler et al., 2007). Since the Mandarin learning requires detecting precise single tone changes it could explain why fundamental pitch listeners seem to be better equipped for acquiring tone languages.

The MANOVA and discriminant analysis revealed that professional musicians outperformed the amateurs and non-musicians in all Mandarin tasks. In previous investigation, we noted that beside professional musicians also amateurs performed better at the acquisition of new non-tone languages. This suggests that little musical training facilitates the learning of new non-tone languages, but higher musical skills may be required for non-tone language speakers to have an advantage in the learning of tone languages such as Mandarin.

The study has also limitations and the singing and pronunciation tasks were rated subjectively. Even though research has shown that subjective and objective rating scales of music performance provide similar information when aspects are carefully introduced (Larrouy-Maestri et al., 2013), there is no equivalent research for language pronunciation tasks available which should be subject to future studies.

### Implications for Pedagogies

The findings of this study have also pedagogical implications and suggest the following aspects. We suggest that the singing benefit is based on enhanced sensorimotor ability and melody as mnemonic (Christiner et al., 2022). The first being crucial to mimic and imitate new language stimuli and the second to retain and memorize utterances. However, these two aspects need to be differentiated from precision as is required for the acquisition of particular language features such as syllable tones in Mandarin. Singing has often been employed as a tool to memorize utterances and to facilitate pronunciation. However, acquiring Mandarin syllable tones demands high tonal precision which becomes neutralized and altered when Mandarin is sung. In this respect, we want to stress that singing ability has to be differentiated from educational programs which make use of singing as a tool to learn new words. We propose that the ability to sing enhances language ability. However, our findings do not indicate that Mandarin syllable tones should be learnt by singing. Instead, the acquisition of syllable tones in Mandarin may be best supported by training of basic auditory skills such as discriminating tones (e.g., high vs. low tones as the Frequency measurement) or by acquiring musical instruments. Research has provided evidence that piano playing enhances neural processing and sound perception of pitch which in turn improved Mandarin word discrimination ability (Nan et al., 2018). In our study, we also detected that the professional musicians performed better than the amateurs and non-musicians in all Mandarin measures—a finding which has also been reported recently (Gottfried, 2019). In addition, research has also shown that musical practice may induce a shift from spectral toward fundamental pitch perception (Seither-Preisler et al., 2007). The latter being better at Mandarin performance according to our finding. Therefore, it can be suggested that learners of Mandarin will acquire syllable tones in Mandarin more easily when they train a musical instrument or basic auditory skills (e.g., tone discrimination tasks).

For STM capacity, it is more difficult to give suggestions. While research has shown that complex working memory paradigms can be improved by training (Klingberg et al., 2002), there is hardly any evidence for capacity changes in verbal STM after extensive practice (Norris et al., 2019). Training of digit spans has not been shown to substantially improve the capacity of verbal STM (Norris et al., 2019). As STM capacity also defined as a language aptitude component (Cain et al., 2004; Robinson, 2005, 2019; Dörnyei, 2006; Wen and Skehan, 2011; Christiner, 2018), one explanation why STM training has been reported to have little effect on language capacity could be that STM capacity may be more of an early acquired or inherent ability. In how far, musical training improves STM capacity and in turn language functions have largely been ignored as far as we know.

## Conclusion

The findings of this investigation have revealed that STM ability, tone frequency, pitch perception preference, singing ability, and musical status were the best predictors for explaining individual differences in Mandarin ability. While STM capacity, tone frequency, and musical status as crucial aspects for Mandarin learning have already been discussed in detail in the current literature, fewer studies have discussed the role of singing in Mandarin. Singing ability was able to explain individual differences in the variability of Mandarin performance. In addition, participants who sang more often during childhood also performed better at Mandarin pronunciation. As far as known, fundamental pitch and spectral listening have not been investigated in the context of Mandarin ability. Results have shown that fundamental pitch listeners indeed seem to be advantaged in tone language learning. Thus, our findings also have implications on educating Mandarin to non-tone language speakers. Since singing predicts individual differences in Mandarin performances, singing tasks, or focusing on melodic aspects of Mandarin may be beneficial for Mandarin learning in initial learning settings. This may facilitate Mandarin pronunciation and retrieval. In addition, we suggest on the basis of our present findings that musical training, such as tone discrimination tasks, should be part of educational programs for non-tone language speakers learning tone languages.

Future research should also focus on the role of STM capacity and musical ability in more detail. While STM capacity and language functions have been studied in detail, STM capacity and musical ability are underrepresented. In addition, there are studies needed which treat STM capacity as mediator between musical ability and language capacity as well as studies should focus on whether musical training improves STM capacity and in turn language ability.

## Data Availability Statement

The original contributions presented in the study are included in the article, further inquiries can be directed to the corresponding author.

## Ethics Statement

The studies involving human participants were reviewed and approved by Medical Faculty of Heidelberg S-778/2018. The patients/participants provided their written informed consent to participate in this study.

## Author Contributions

MC and JR developed the Mandarin measures, contributed to the conception and design of the work, and drafted the work. MC, CG, JB, and PS were involved in the acquisition of data. AS-P and MC performed the statistical analysis. MC was responsible for finalizing the work. CG and AS-P performed a critical revision of the manuscript. All authors contributed to the article and approved the submitted version.

## Funding

MC is funded within the Post-DocTrack Program of the OeAW. Open Access Funding by the University of Graz.

## Conflict of Interest

The authors declare that the research was conducted in the absence of any commercial or financial relationships that could be construed as a potential conflict of interest.

## Publisher’s Note

All claims expressed in this article are solely those of the authors and do not necessarily represent those of their affiliated organizations, or those of the publisher, the editors and the reviewers. Any product that may be evaluated in this article, or claim that may be made by its manufacturer, is not guaranteed or endorsed by the publisher.
