# Supplementary material for: Singing Mandarin? What Short-Term Memory Capacity, Basic Auditory Skills, and Musical and Singing Abilities Reveal About Learning Mandarin
Source: Front Psychol. 2022 Jun 16;13:895063. doi: 10.3389/fpsyg.2022.895063 (PMC9245042; doi:10.3389/fpsyg.2022.895063)
Supplement: Supplementary file 1 [file Table_1.DOCX]

Supplementary Material

# Supplementary Data

The supplement contains relevant background information and additional statistical material for further illustration.

# Interrater reliability Mandarin and Singing

For assessing the reliability of the ratings of the performances intraclass correlation coefficients were applied for the Mandarin and singing ratings. The ratings of all five Mandarin raters for the three sentences were entered and a two-way mixed effects model, in which people effects are random and measures effects are fixed, were performed. The same approach was applied for the singing ratings. There the ratings of the four singing experts were entered. The results have shown that the ratings were reliable and above the accepted value of 0.7 (Koo & Li, 2016). Table S2 below shows the results of the intraclass correlation coefficient of the Mandarin ratings, while table S3 illustrates the intraclass correlation coefficient of the singing ratings.

Table S1. Intraclass correlation coefficients Mandarin.

|  |  | 95% Confidence Interval | |  | F Test with True Value 0 | | | |
| --- | --- | --- | --- | --- | --- | --- | --- | --- |
|  | Intraclass Correlation | Lower Bound | Upper Bound |  | Value | df1 | df2 | Sig |
| Average Measures | .793 | 0,709 | 0,854 |  | 7,398 | 107 | 1498 | 0,000 |

Table S1 illustrates the intraclass correlation coefficient of the Mandarin ratings.

Table S2. Intraclass correlation coefficients Singing.

|  | | | | | | | | |
| --- | --- | --- | --- | --- | --- | --- | --- | --- |
|  |  | 95% Confidence Interval | |  | F Test with True Value 0 | | | |
|  | Intraclass Correlation | Lower Bound | Upper Bound |  | Value | df1 | df2 | Sig |
| Average Measures | .881 | .833 | .917 |  | 9,403 | 107 | 321 | 0,000 |

Table S2 illustrates the intraclass correlation coefficient of the singing ratings.

# Concept Singing behaviour during childhood

For the interrater reliability of the singing behaviour during childhood concept, Cronbach's α coefficients were calculated. Results have shown that the reliability is high, all Cronbach’s α =.79. Since all questions were above the statistically accepted range of 0.7 (Field, 2009), none of the questions were deleted. The eight questions and the Cronbach's α are provided below (see table S3 below).

| Table S3. Concept Singing behaviour during childhood. | |
| --- | --- |
|  | Cronbach's α if item deleted |
| As a child I enthusiastically joined in with the singing at church and similar events whenever the possibility arose (Q1). | .778 |
| As a child I was encouraged to sing by my caretakers and we sang together on a weekly basis even if there were no special events. (Q2). | .801 |
| As a child I enjoyed singing in a choir, with friends, at Christmas, birthdays, or at similar occasions (Q3). | .756 |
| As a child I sang very often since I wanted to become a musician or singer (Q4). | .780 |
| As a child I used to sing whenever I could such as in the bathroom, in the car, when I played with friends (Q5). | .761 |
| As a child I liked being a member of our school choir, or would have liked being a member or a school choir (Q6). | .742 |
| As a child I enjoyed singing a song that had been played to me (e.g., in the radio) (Q7). | .789 |
| As a child I used to sing more often than my friends (Q8). | .790 |

Note that the questions were translated.

# Regression models of the three Mandarin variables

Three multiple linear regression models were performed where each of the three Mandarin variables were the dependent variable. The independent variables were entered in the multiple linear regression models only if a probability of F-change < 0.05 was given. The predictors are provided below.

Table S4. Regression models of the three Mandarin tasks

| *Multiple regression models explaining the variance in Mandarin S+D+P* | | |
| --- | --- | --- |
|  |  |  |
| **Predictor** | **Partial correlation (pr)** | ***p-*Value** |
|  |  |  |
| Dependent variable: Mandarin S |  |  |
| *R* = 0.45, *F*(1, 104) = 7.41, *p* = 0.008 | |  |
| Musical status | 0.27 | 0.006 |
| STM | 0.26 | 0.008 |
| Dependent variable: Mandarin D |  |  |
| *R* = 0.52, *F*(1, 103) = 4.58, *p* = 0.035 | |  |
| STMF | 0.38 | < 0.001 |
| Pitch PP | -0.24 | 0.020 |
| Musical status | 0.22 | 0.035 |
| Dependent variable: Mandarin P |  |  |
| *R* = 0.56, *F*(1, 102) = 4.21, *p* = 0.043 | |  |
| Pitch PP | -0.35 | < 0.001 |
| S childhood | 0.35 | < 0.001 |
| STM | 0.28 | 0.005 |
| Frequency | -0.20 | 0.043 |

# Reference List

Field, A. (2009). Discovering statistics using SPSS (3. ed.). Los Angeles, CA: Sage.

Koo, T. K., & Li, M. Y. (2016). A Guideline of Selecting and Reporting Intraclass Correlation Coefficients for Reliability Research. Journal of chiropractic medicine, 15(2), 155–163.

Yang, J. (2009). Hanyu jiaocheng ([Nachdr.]). Beijing: Beijing Language and Culture Univ. Press.
